# Phenotypic Alterations Involved in CD8+ Treg Impairment in Systemic Sclerosis

**DOI:** 10.3389/fimmu.2017.00018

**Published:** 2017-01-19

**Authors:** Simone Negrini, Daniela Fenoglio, Alessia Parodi, Francesca Kalli, Florinda Battaglia, Giorgia Nasi, Monica Curto, Samuele Tardito, Francesca Ferrera, Gilberto Filaci

**Affiliations:** ^1^Center of Excellence for Biomedical Research, University of Genoa, Genoa, Italy; ^2^Department of Internal Medicine, Clinical Immunology Unit, University of Genoa, Genoa, Italy

**Keywords:** systemic sclerosis, scleroderma, CD8+ T regulatory cells, CD127, CD39

## Abstract

Systemic sclerosis (SSc) is a connective tissue disease characterized by tissue fibrosis, vasculopathy, and autoimmunity. Although the exact pathogenetic mechanisms behind SSc remain to be fully elucidated, a great deal of evidence suggests the existence of an unbalanced ratio between the effector and regulatory arms of the immune system. With regard to the T regulatory (Treg) compartment, we observed that CD8+ Treg subsets display functional defects in SSc-affected patients. Since CD127 down-modulation and CD39 upregulation have been observed on Treg subsets, the phenotypic expression of these molecules was analyzed on the CD8+CD28− Treg precursors and on CD8+ Treg cells generated *in vitro* through interleukin-10 commitment. Immunophenotypic data from SSc patients were compared to those obtained from healthy subjects. The analyses performed on *ex vivo*-isolated CD8+CD28− Treg precursors did not show any significant differences in CD39 or CD127 expression as compared to values obtained from healthy donors. On the contrary, *in vitro*-generated CD8+ Tregs obtained from SSc patients displayed reduced expression of the CD39 molecule as compared to controls. Moreover, the percentage of CD127+ cells was significantly higher in *in vitro*-generated CD8+ Tregs from SSc patients compared to CD8+ Tregs obtained from healthy donors. Taken together, these findings may indicate an impairment of maturation processes affecting CD8+ Treg cells in SSc patients. This impairment of maturation involves phenotypic alterations that are mainly characterized by a deficient CD39 upregulation and a lack of down-modulation of the CD127 molecule.

## Introduction

Several subsets of T regulatory (Treg) lymphocytes with distinct phenotypes and mechanisms of action have been described among CD8+ T cells. As the CD4+ Treg counterpart, these cells play an important role in physiological and pathological conditions such as autoimmune, infectious, or neoplastic diseases (1, 2). In particular, our group has characterized a subset of CD8+ Treg lymphocytes that mediate suppression without antigen restriction. These CD8+ Treg cells originate from circulating CD8+CD28−CD127+CD39− T lymphocytes through *in vitro* conditioning with interleukin (IL)-2 and IL-10; therefore, they belong to the “peripherally induced” CD8+ Treg subpopulations (1, 3, 4). From a functional point of view, these non-antigen-specific CD8+ Tregs inhibit both T cell proliferation and cellular cytotoxicity through the secretion of cytokines, mostly IL-10, and do not require direct cell-to-cell contact to mediate their inhibitory functions (1, 5, 6). Furthermore, we recently identified an additional regulatory mechanism involving the activity of CD39 ecto-nucleotidase (4), as already described for CD4+ Tregs. Finally, these cells share with CD4+ Treg the down-modulation of the CD127 molecule, so that their exact phenotype is CD8+CD28−CD127loCD39+ (3, 4, 7).

Systemic sclerosis (SSc) is a systemic connective tissue disease characterized by small vessel vasculopathy, immune alterations, and fibroblast dysfunction leading to diffuse tissue fibrosis (8). Even though SSc pathogenesis is still largely elusive, autoimmunity seems to be implicated in disease development as suggested by the presence of several abnormalities in humoral and cellular immunity (9). Concerning this latter point, alterations of the normal functional balance between pro-inflammatory subpopulations, in particular Th17, and Treg subpopulations have been demonstrated in patients affected by SSc (10–15).

With regard to the Treg compartment, we observed that both CD4+ and CD8+ Treg subsets show quantitative and functional defects in SSc patients (16). This is reminiscent of what we recently found studying CD8+ Tregs in primary biliary cirrhosis (PBC), an organ-specific, fibrotic autoimmune disease that share with SSc several pathogenetic mechanisms (17). In PBC, CD8+ Treg abnormalities seem to correlate with increased CD127 antigen expression and reduced CD39 molecule expression. This observation is not surprising since CD127, the α-chain of the IL-7 receptor, is mainly expressed on effector cells and is physiologically down-modulated on Treg lymphocytes (18), while CD39 is a membrane-bound nucleosidase whose activity has been correlated with Treg function (4, 7, 19).

Therefore, we decided to analyze CD127 and CD39 molecule expression in CD8+ Tregs derived from SSc patients in order to determine whether alterations affecting these two pathways could explain the functional impairment observed in the CD8+ Treg subpopulation.

## Materials and Methods

### Patients and Controls

Twenty-eight patients affected with SSc (mean age 64 ± 14 years, female/male ratio 3/1) were enrolled at the Division of Internal Medicine and Clinical Immunology of the Department of Internal Medicine, University of Genoa, after providing informed consent.

Diagnosis of SSc was made according to the American College of Rheumatology criteria (20). Enrolled patients were being treated with vasoactive (e.g., iloprost or dihydropyridine calcium channel blockers) but not with immunosuppressive drugs. Ten healthy subjects, age and sex matched with the SSc patients, were enrolled as controls.

The study was carried out in compliance with the Helsinki Declaration and was approved by the Ethics Committee of the San Martino Hospital in Genoa, Italy.

### Monoclonal Antibodies (mAbs)

The following mAbs were used for immunostaining and analysis by flow cytometry (FACS): allophycocyanin (APC)-cyanin 7 (Cy7) conjugated anti-CD4 (Clone RPA-T4), APC-conjugated anti-CD39 (Clone TU66), phycoerythrin-conjugated anti-CD127 (Clone M21), PeCy7 conjugated anti-CD25 (Clone M-A251), fluorescein isothiocyanate-conjugated anti CD45RA (Clone HI100), Brilliant Violet 421-conjugated anti-CD8 (Clone RPA-T8), and Horizon V500-conjugated anti-CD3 (Clone UCHT1) [Becton Dickinson (BD) Biosciences]; PerCP-cyanin 5-conjugated anti-CD28 (Clone CD28.2) (Biolegend). CD8+ T cells (1 × 10^6^) were incubated with a mAb cocktail for 30 min at 4°C in the dark. After staining procedures, the samples were washed with PBS and stored at 4°C. The samples were acquired and analyzed by a FACSCanto flow cytometer II equipped with three lasers (BD Biosciences, San Josè, CA, USA) using the FACSDIVA software (BD Biosciences). Fluorescence minus one was used to properly interpret flow cytometry data.

### Purification of CD8+ T Lymphocytes

Peripheral blood mononuclear cells (PBMCs) were purified from the venous heparinized blood samples of healthy controls and SSc patients by centrifugation on Ficoll-Hypaque gradient (Biochrom AG, Berlin, Germany) for 30 min at 1,800 rpm. CD8+ T lymphocytes were isolated by sequential cycles of cell sorting on magnetic beads using microbeads conjugated with a mAb specific for the CD8 antigen (Dynal CD8 positive isolation kit, Invitrogen by Life Technologies Ltd., Paisley, UK) following the manufacturer’s instructions.

### *In Vitro* Generation of CD8 Tregs from the Peripheral Blood Precursors

CD8+ Tregs were generated as previously described (21). Briefly, purified CD8+ T lymphocytes (2 × 10^5^ cells/well) resuspended in culture medium consisting of RPMI 1640 culture medium (Gibco by Life Technologies Ltd., Paisley, UK) added with 10% fetal calf serum (Invitrogen by Life Technologies Ltd., Paisley, UK) were incubated with 20 U/ml of IL-2 (Proleukin, Eurocetus, Amsterdam, The Netherlands) and 10 ng/ml of IL-10 (PeproTech, Rocky Hill, NJ, USA) in 96-well flat bottomed plates (Sardsted) at 37°C for 7 days. The regulatory activity and the membrane antigen study were performed on both freshly *ex vivo*-purified CD8+ T cells and on *in vitro*-generated CD8+ Tregs.

### Proliferation Suppression Assay

The suppressive activity of Treg was evaluated by monitoring the inhibition of dye dilution in PBMC stained with carboxyfluorescein diacetate succinimidyl ester (CFDA-SE, 5 µM, Molecular Probes, Invitrogen).

Briefly, the PBMC-CFDA-SE+ were pulsed with the anti-CD3 UCTH-1 mAb (5 µg/ml, BD Bioscience) and cultured for 5 days in a 96-well U bottomed plate (1 × 10^5^ cells/well) in the presence (or not) of *ex vivo*- or *in vitro*-generated CD8+ T lymphocytes (1 × 10^5^ cells/well). Then, the samples were harvested, washed in PBS, and analyzed by flow cytometry. The dead cells were excluded from analysis by adding 7-aminoactinomycin D (7-AAD) (BD Bioscience) prior to analysis. Suppression activity was expressed as the percentage reduction of the proliferation in the presence of CD8+ Treg lymphocytes compared to the levels of proliferation observed in control cultures of PBMCs cultured in the absence of Treg cells. A suppression activity ≥25% was considered significant. This threshold was chosen based on the results achieved in a large historical cohort of more than 50 healthy subjects of both sexes with age ranging from 18 to 87 years. In healthy donors, CD8+ Treg suppression activity never fell below 25%.

### Statistical Analyses

Statistically significant differences between frequencies were analyzed by Fisher’s exact test. Statistically significant differences between mean values were analyzed by the Mann–Whitney test for non-parametric values. Differences were considered statistically significant when *p* < 0.05. Statistical analyses were performed using GraphPad Prism version 6.03 for Windows (GraphPad Software, San Diego, CA, USA).

## Results

### Altered Generation of CD8 Tregs in SSc Patients

**Figure 1:**
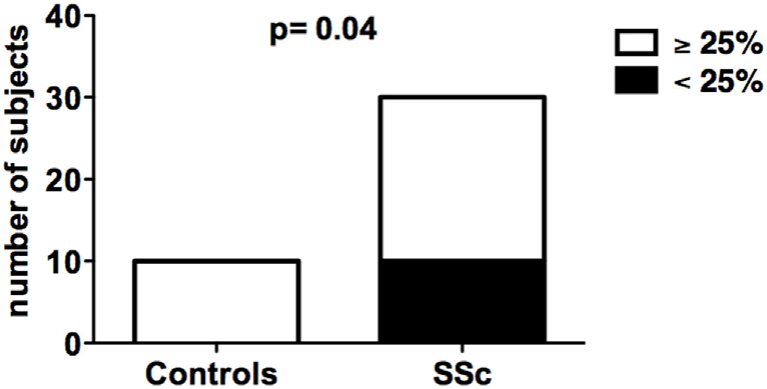
***In vitro* generation of CD8+ T regulatory (Treg) cell in healthy donors and systemic sclerosis (SSc) patients**. The bars indicate the frequency of healthy donors (left bar) and SSc patients (right bar) showing normal (suppression activity ≥25%, white bars) or impaired (suppression activity <25%, black bars) CD8+ Treg function. Statistical analyses were performed by Fisher’s exact test.

The suppressive activity of *in vitro*-generated CD8+ Tregs obtained from SSc patients was analyzed in comparison with that of healthy donors. *In vitro* generation of CD8+ Tregs led to the differentiation of circulating CD8+ T cells into CD8+CD28−CD127−CD39+ T lymphocytes mediating a suppressive activity ≥25% in suppression assays in 10 out of 10 (100%) healthy donors. In contrast, impaired *in vitro* generation of CD8+ Tregs was observed in 1/3 of SSc patients since *in vitro*-generated CD8+ Tregs from 10 out of 28 SSc patients failed to exert a suppressive activity above the 25% threshold. The differences between the frequencies of subjects showing ≥25% suppressive activity by their CD8+ Treg in healthy controls and SSc patients was statistically significant (*p* = 0.04) as assessed by Fisher’s exact test (Figure 1; Tables S1 and S2 in Supplementary Material).

### CD39 Expression on CD8+ Treg Cells

Given that the expression of CD39 is related to the suppressive activity of both CD4 and CD8 Treg cells (4, 7), its expression was analyzed on freshly isolated CD8+CD28− (formally CD8+ Treg precursors) and on *in vitro*-generated CD8+ Treg cells from both SSc patients and healthy donors.

**Figure 2:**
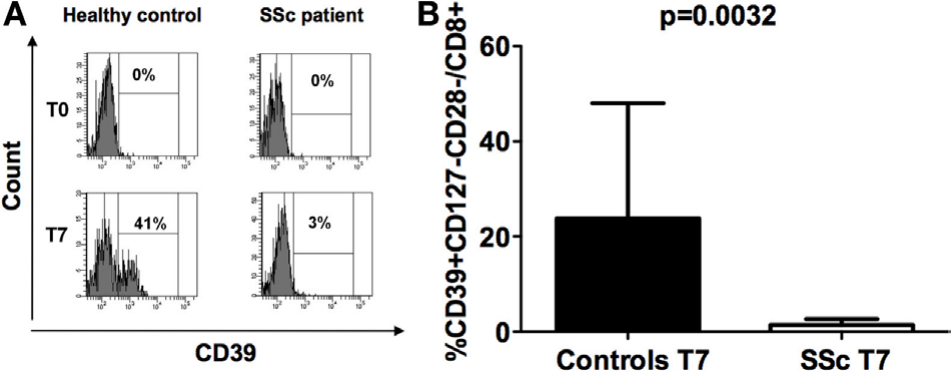
**CD39 expression on freshly isolated CD8+CD28− lymphocytes and *in vitro*-generated CD8+ T regulatory (Treg) cell**. **(A)** Histograms showing the percentages of expression of the CD39 molecule on freshly isolated CD8+CD28− lymphocytes (T0) and on interleukin-10-committed CD8+CD28−CD127− T cells (T7) from a representative healthy donor (left) and a representative systemic sclerosis (SSc) patient (right). **(B)** Mean percentages of CD39+CD8+CD28− Treg cells in healthy donors (black bar) and SSc patients (white bar). Statistical analyses were performed by Mann–Whitney test.

CD39 molecule was undetectable (with values below 0.01% among the CD3+ T cell population) on freshly isolated CD8+CD28− lymphocytes obtained from both SSc patients and healthy controls at T0 (Figure 2A). Interestingly, while CD39 expression was strongly upregulated in CD8+ Tregs generated from healthy subjects with respect to their circulating precursor CD8+CD28− T cells, this increase was not observed in SSc patients. Indeed, CD39 expression was significantly lower in *in vitro*-generated CD8+ Tregs from SSc patients with respect to *in vitro*-generated CD8+ Tregs from healthy donors, as assessed by Mann–Whitney test (*p* = 0.0032; Figures 2A,B).

### CD127 Expression in CD8 Treg Subpopulation

We previously observed that CD127 antigen down-modulation is strictly related to the correct functional maturation/generation of CD8+ Tregs: thus, we explored its expression on freshly isolated CD8+CD28− and on *in vitro*-generated CD8+ Tregs from SSc patients and healthy donors.

**Figure 3:**
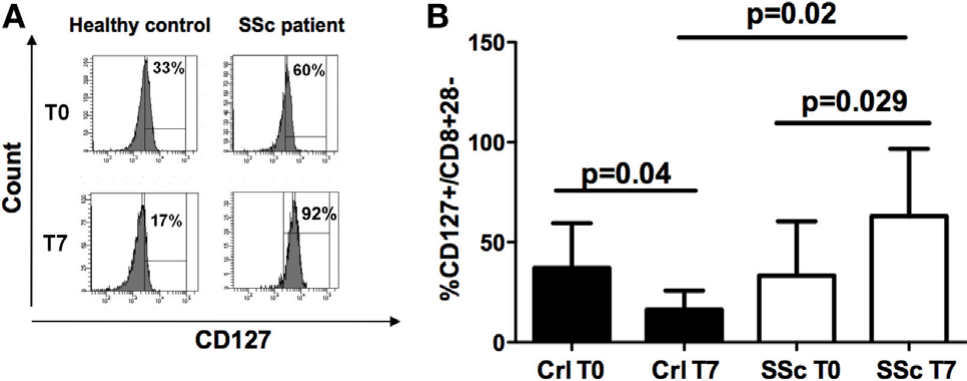
**CD127 expression on freshly isolated CD8+CD28− lymphocytes and *in vitro*-generated CD8+ T regulatory (Treg) cell**. **(A)** Mean percentages of CD127+ cells in freshly isolated CD8+CD28− lymphocytes (T0) and *in vitro*-generated CD8+ Treg (T7) in healthy donors (black bar) and systemic sclerosis (SSc) patients (white bar). **(B)** Histograms showing the percentages of expression of the CD127 molecule on freshly isolated CD8+CD28− lymphocytes (T0) and on interleukin-10-committed CD8+CD28− T cells (T7) from a representative healthy donor (left) and a representative SSc patient (right). Statistical analyses were performed by Mann–Whitney test.

No differences in CD127 expression were observed on CD8+CD28− cells from the peripheral blood of SSc patients and healthy controls at T0 (Figures 3A,B). However, CD127 expression diminished on CD8+CD28− after IL10 commitment for Treg generation in healthy donors (*p* = 0.04), while it increased on the corresponding cells from SSc patients (*p* = 0.029) with respect to their circulating precursors (Figures 3A,B, analyses performed by Mann–Whitney test). Accordingly, the percentage of CD127+ T cells on CD8+CD28− T lymphocytes after *in vitro* CD8+ Treg generation was significantly higher in cells from SSc patients as compared to healthy subjects (*p* = 0.02, Figure 3A).

**Figure 4:**
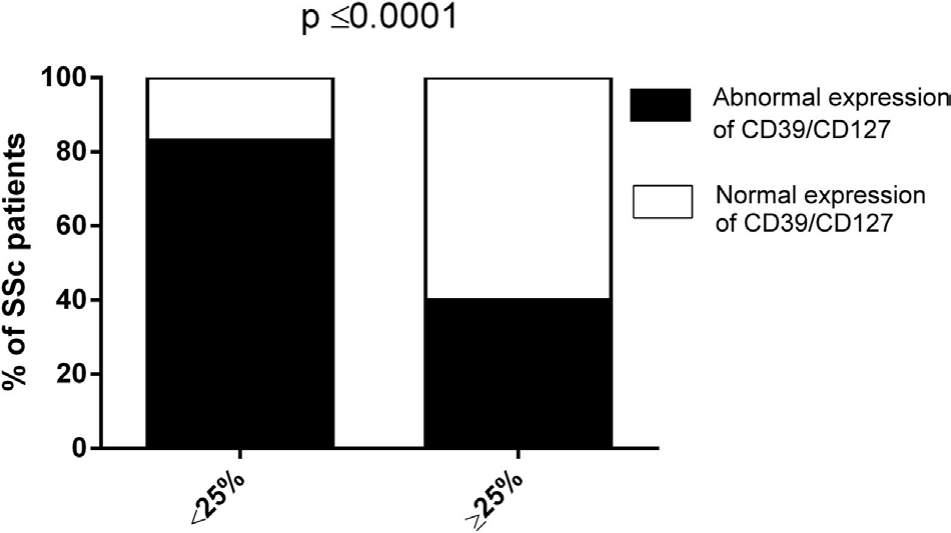
**Analysis of the suppression activity of CD8+ T regulatory (Treg) cell generated from systemic sclerosis (SSc) patients in relation to the expression patterns of CD39/CD127**. The bars display the frequencies of SSc patients showing impaired (suppression activity <25%, left bar) or normal (suppression activity ≥25%, right bar) CD8+ Treg function in relation to the expression patterns of CD39/CD127 molecules (normal—white bars, abnormal—black bars). Statistical analyses were performed by Fisher’s exact test.

Collectively, our data envisage the dependence of abnormal CD8+ Treg function in SSc patients on their altered expression of CD39 and/or CD127 molecules. In order to find support to this pathogenic mechanism, the frequencies of SSc patients showing impaired (suppression activity <25%) or normal (suppression activity ≥25%) CD8+ Treg function were related, by Fisher’s exact test analysis, to the existence, in the CD8+ Treg of each single patient, of an abnormal expression of CD39 and/or CD127 molecules (considering as altered expression the downregulation of CD39 and the upregulation of CD127 with respect to the relative CD8+CD28− T cell precursors, respectively). Figure 4 and Table 1 show that the frequency of SSc patients showing abnormal expression of CD39 and/or CD127 molecules within patients with altered CD8+ Treg function was more than twice that observed in patients with normal CD8+ Treg activity. This observation highlights the strict relationship between altered CD39 and/or Cd127 expression and CD8+ Treg function.

**Table 1:** **Percentage of CD127 and CD39 expression on CD8+CD28− T cells before and after *in vitro* CD8+ Treg generation**.

Patient No.	CD8+ Treg suppression (< or ≥25%)	CD127+/CD8+CD28− T cells at T0^a^ (%)	CD127+/CD8+CD28− T cells at T7^b^ (%)	CD39+/CD8+CD28− T cells at T0^a^ (%)	CD39+/CD8+CD28− T cells at T7^b^ (%)
1	≥	13	93	1	1.7
2	<	17	81	2	1.5
3	<	12	11	1	1.2
4	<	19	89	1.5	0.3
5	≥	33	29	0.6	0.8
6	≥	20	35	2.2	4.6
7	≥	40	36	2.2	2.4
10	≥	9.4	25	1.3	3
11	≥	27	2	0	0.5
12	≥	83	80	0.3	1
13	≥	80	85	0.3	1
14	≥	60	55	0	1
15	<	30	35	0.5	0
16	<	58	64	0.5	0
17	≥	60	50	1	1.4
18	≥	19	26	2	0.1
19	<	33	30	0.1	0.5
20	≥	82	78	0.1	1
21	≥	40	99	0	1
22	≥	15	10	0.1	1
23	≥	20	30	1	0.5
24	≥	40	30	0.5	1
25	≥	15	10	0	0.5
26	≥	20	15	0.5	1
27	<	15	35	0.5	0.1
28	<	19	22	1.6	0
29	<	27	25	1	0
30	<	25	20	0.7	0

*^a^Baseline*.

*^b^At the end of 7 days in vitro generation of CD8+ Treg*.

## Discussion

The results of our work show that (1) *in vitro* generation of CD8 Treg lymphocytes may be altered in SSc patients, thus leading to impaired suppressor activity; (2) CD8 Tregs generated from SSc patients display reduced CD39 expression and increased CD127 expression compared to CD8+ Tregs generated from healthy donors.

Systemic sclerosis is a chronic connective tissue disease characterized by diffuse tissue fibrosis, microangiopathy, and immune abnormalities. Although the cause of SSc is unknown, autoimmunity seems to play an important role in the pathogenesis of the disease. Support for this hypothesis includes the presence of autoantibodies, evidence of abnormalities in B and T cell compartments in both the circulation and the affected organs, altered levels of growth factors, chemokines and cytokines, and the potential clinical overlap with other clearly recognized autoimmune diseases such as systemic lupus erythematosus and rheumatoid arthritis (8, 17, 22, 23). Our group recently described the existence of an imbalance between effector and regulatory responses in SSc patients. In particular, we observed that an increased Th17 effector response is associated with defective T cell regulation involving quantitative and functional alterations in both CD4+CD25+ and CD8+ Treg subpopulations (16). Furthermore, we recently described the qualitative defects affecting CD8+ Tregs in PBC, an organ-specific, fibrotic autoimmune disease. In this disease, CD8+ Treg functional abnormalities correlate with the defective expression of the CD39 molecule and the increased expression of the CD127 antigen (17).

CD39 is a cell surface ecto-nucleotidase that hydrolyzes extracellular ATP or ADP to AMP. Additionally, in concert with CD73, another ecto-nucleotidase which dephosphorylates AMP, its activity leads to the production of adenosine. Furthermore, there is increasing evidence indicating that CD39 and purinergic signaling alterations may be implicated in pathological conditions such as neoplastic and inflammatory diseases (24). Although the role of CD39 has been less investigated in CD8+ Tregs, our group recently reported that CD39 is expressed and involved in the activity of non-antigen-specific CD8+ Treg cells (4). Furthermore, we correlated the defective expression of CD39 molecules with the functional impairment of CD8+ Tregs generated from patients affected by PBC (17). Based on this, we explored the role of CD39 in SSc. Interestingly, CD8+ Tregs generated from healthy subjects exhibit a significant upregulation of CD39 expression with respect to their precursor CD8+CD28− T cells, while this phenomenon was not observed in CD8+ Tregs generated from SSc patients. Considering the important role of CD39 for Treg regulatory activity, CD39 expression defects could explain, at least in part, the functional impairment observed in CD8+ Tregs generated from SSc patients.

To further define if additional phenotypic abnormalities could be involved in functional alterations found in CD8+ Tregs generated from SSc patients, we analyzed the role of CD127. CD127, the IL-7 receptor α chain, is reportedly a useful marker for identifying memory and effector T cells, while its expression is down-modulated on CD4+CD25+ Treg cells (18, 25, 26).

Similarly to what we observed for CD4+CD25+ Tregs, we previously demonstrated that maturation of circulating CD8+CD28− T cells to CD8+ Tregs is associated with CD127 down-modulation (3). CD127 expression on CD8+CD28− cells from the peripheral blood of SSc patients was similar to what was observed in healthy controls. As expected, CD8+ Tregs generated from healthy donors displayed a down-modulation of CD127 expression with respect to their circulating precursors. On the contrary, CD127 expression increased in CD8+ Tregs generated from SSc patients. Our observation is consistent with recent literature data indicating that IL-7 may play a significant role in numerous T cell-driven chronic inflammatory autoimmune diseases, and that this cytokine likely regulates the proliferation of autoreactive T cells (27).

Our data show for the first time that CD8+ Treg lymphocytes derived from SSc patients display a maturation defect that is characterized by reduced CD39 expression associated with abnormal CD127 overexpression. Alterations of CD39 and CD127 expression could explain, at least partly, the functional defects observed in the CD8+ Tregs generated from patients affected by SSc.

## Author Contributions

All the authors listed have made substantial, direct, and intellectual contribution to the work and approved it for publication.

## Conflict of Interest Statement

The authors declare that the research was conducted in the absence of any commercial or financial relationships that could be construed as a potential conflict of interest.

## Supplementary Material

Table S1 **Raw data of percent suppression activity by CD8+ Treg generated from 10 healthy donors**.

Table S2 **Raw data of percent suppression activity by CD8+ Treg generated from 28 SSc patients**.

Figure S1 **Percentage expression of CD39 (A) and CD127 (B) molecules on CD8+CD28+ T cells**. Panel **(A)** also shows CD39 percentage expression on freshly isolated CD4+ Treg. T0: analyses performed on freshly purified cells; T7: analyses performed after 7 days incubation with IL2 and IL10 for CD8+ Treg generation. HC, healthy controls; SSc, systemic sclerosis patients.
