# Supplementary material for: Phenotypic Alterations Involved in CD8+ Treg Impairment in Systemic Sclerosis
Source: Front Immunol. 2017 Jan 19;8:18. doi: 10.3389/fimmu.2017.00018 (PMC5243838; doi:10.3389/fimmu.2017.00018)
Supplement: Table S1 — Raw data of percent suppression activity by CD8+ Treg generated from 10 healthy donors. [file Table_1.DOCX]

| **Supplementary Table 1. Raw data of percent suppression activity by CD8+ Treg generated from 10 healthy donors.** | |
| --- | --- |
| **HD* N.** | **% suppression activity** |
| 1 | 70 |
| 2 | 67 |
| 3 | 57 |
| 4 | 47 |
| 5 | 59 |
| 6 | 50 |
| 7 | 76 |
| 10 | 39 |

*HD: healthy donors.
