# Supplementary material for: Phenotypic Alterations Involved in CD8+ Treg Impairment in Systemic Sclerosis
Source: Front Immunol. 2017 Jan 19;8:18. doi: 10.3389/fimmu.2017.00018 (PMC5243838; doi:10.3389/fimmu.2017.00018)
Supplement: Table S2 — Raw data of percent suppression activity by CD8+ Treg generated from 28 SSc patients. [file Table_2.DOCX]

| **Supplementary Table 2. Raw data of percent suppression activity by CD8+ Treg generated from 28 SSc patients.** | |
| --- | --- |
| **Patient N.** | **% suppression activity** |
| 1 | 51 |
| 2 | 17 |
| 3 | 0 |
| 4 | 0 |
| 5 | 85 |
| 6 | 75 |
| 7 | 79 |
| 10 | 88 |
| 11 | 78 |
| 12 | 81 |
| 13 | 83 |
| 14 | 70 |
| 15 | 0 |
| 16 | 0 |
| 17 | 79 |
| 18 | 62 |
| 19 | 22 |
| 20 | 52 |
| 21 | 87 |
| 22 | 94 |
| 23 | 70 |
| 24 | 41 |
| 25 | 77 |
| 26 | 68 |
| 27 | 20 |
| 28 | 0 |
| 29 | 20 |
| 30 | 0 |
