# Supplementary figures and images for: Phenotypic Alterations Involved in CD8+ Treg Impairment in Systemic Sclerosis
Source: Front Immunol. 2017 Jan 19;8:18. doi: 10.3389/fimmu.2017.00018 (PMC5243838; doi:10.3389/fimmu.2017.00018)

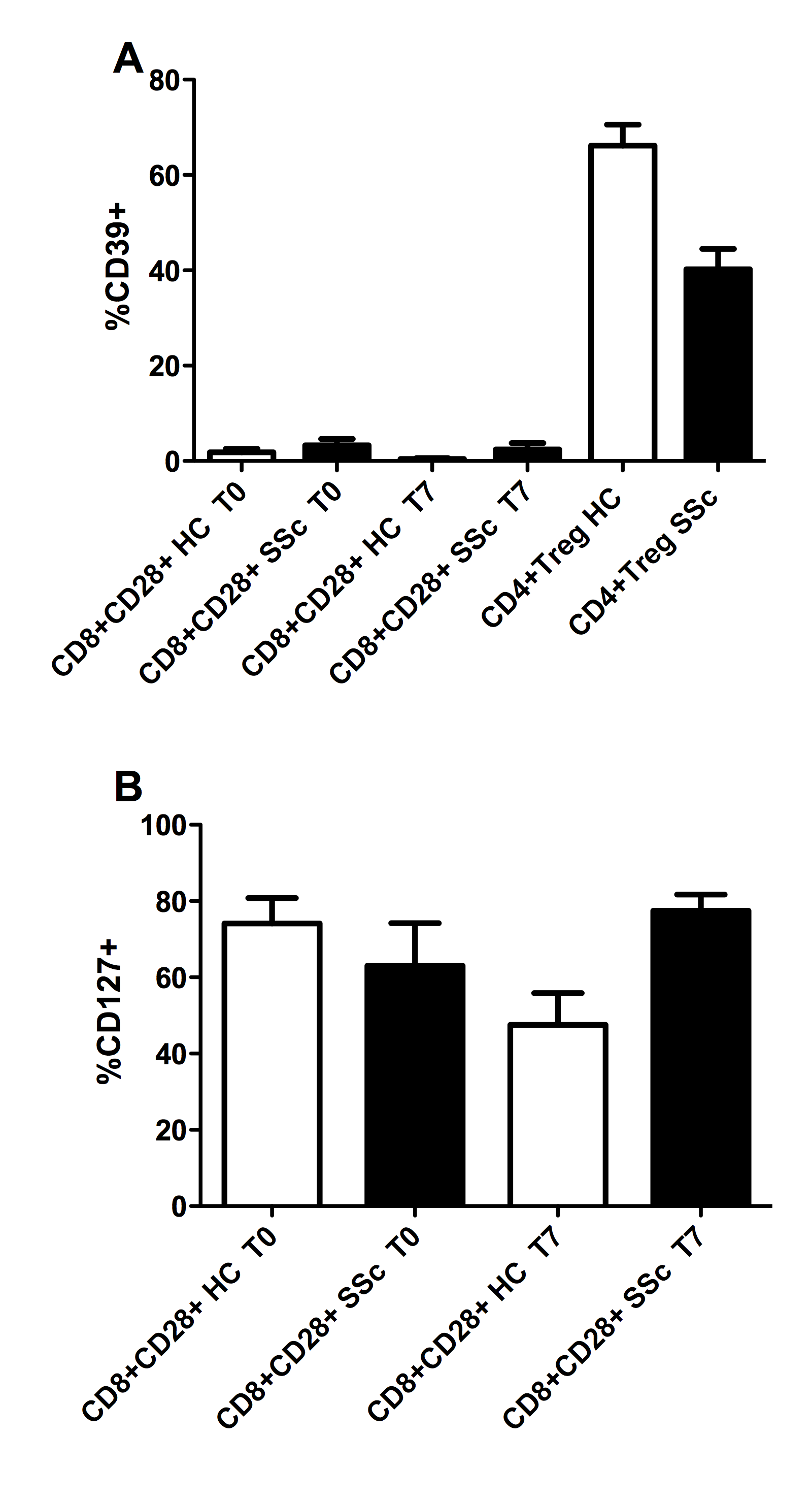

Supplement: Figure S1 — Percentage expression of CD39 (A) and CD127 (B) molecules on CD8+CD28+ T cells. Panel (A) also shows CD39 percentage expression on freshly isolated CD4+ Treg. T0: analyses performed on freshly purified cells; T7: analyses performed after 7 days incubation with IL2 and IL10 for CD8+ Treg generation. HC, healthy controls; SSc, systemic sclerosis patients. [file Image_1.TIFF]
